# Estimating the Confidence Level of White Matter Connections Obtained with MRI Tractography

**DOI:** 10.1371/journal.pone.0004006

**Published:** 2008-12-23

**Authors:** Xavier Gigandet, Patric Hagmann, Maciej Kurant, Leila Cammoun, Reto Meuli, Jean-Philippe Thiran

**Affiliations:** 1 Signal Processing Laboratory (LTS5), Ecole Polytechnique Fédérale de Lausanne (EPFL), Lausanne, Switzerland; 2 Department of Radiology, University Hospital Center and University of Lausanne (CHUV-UNIL), Lausanne, Switzerland; 3 Laboratory for computer Communications and Applications, Ecole Polytechnique Fédérale de Lausanne (EPFL), Lausanne, Switzerland; Indiana University, United States of America

## Abstract

**Background:**

Since the emergence of diffusion tensor imaging, a lot of work has been done to better understand the properties of diffusion MRI tractography. However, the validation of the reconstructed fiber connections remains problematic in many respects. For example, it is difficult to assess whether a connection is the result of the diffusion coherence contrast itself or the simple result of other uncontrolled parameters like for example: noise, brain geometry and algorithmic characteristics.

**Methodology/Principal Findings:**

In this work, we propose a method to estimate the respective contributions of diffusion coherence versus other effects to a tractography result by comparing data sets with and without diffusion coherence contrast. We use this methodology to assign a confidence level to every gray matter to gray matter connection and add this new information directly in the connectivity matrix.

**Conclusions/Significance:**

Our results demonstrate that whereas we can have a strong confidence in mid- and long-range connections obtained by a tractography experiment, it is difficult to distinguish between short connections traced due to diffusion coherence contrast from those produced by chance due to the other uncontrolled factors of the tractography methodology.

## Introduction

Due to its ability to probe the tissue microstructure, Diffusion MRI is known to be a very powerful tool to infer brain anatomical connectivity [1]. Diffusion Tensor Imaging (DTI) [2], which models the diffusion as a first-order tensor, is probably the most used technique to study brain neuronal circuitry. However, due to the limited angular resolution of DTI the interest towards higher angular resolution diffusion MRI methodologies is increasing. One of these methodologies is the Diffusion Spectrum Imaging (DSI) [3], [4], [5], which allows to map the diffusion of water molecules by reconstructing the spectrum of the spin displacement. Let us also mention the existence of other high resolution techniques not discussed in this work, such as Q-ball [6] or spherical deconvolution [7]. The increased interest in Diffusion MRI has led to the development of various tractography algorithms, whose aim consists in inferring from the diffusion measurement the trajectories of the axonal bundles in the brain, allowing the study of the fiber tract architecture. We can divide these numerous tractography methodologies into two main classes, the probabilistic (e.g. [8]–[10]) algorithms providing connection probability maps, and the deterministic ([11]–[13]) algorithms generating virtual fibers.

Diffusion MRI data contain noise that systematically affects the tractography [14], [15], regardless of the method used to generate fiber tracts. Angular and spatial resolution, brain shape and of course the MRI acquisition methodology, as well as the tractography algorithm itself, are potential sources of errors in the mapping of brain connectivity [16]–[18]. This immediately raises the question of validation of the results. There exist several approaches to address this difficult problem. First, the tractography algorithms can be tested on synthetic data, where all the parameters of the underlying model are known [14]. Another approach is to correlate the reconstruction of a small set of tracts with some gold standard methods [14], [19], [20]. These methods are nonetheless partial validation and therefore complementary studies which analyze other aspects of the problem, such as the reliability of computed white matter connections across the whole brain, are welcome. In this study, we address the following question: How do we know that a given tractography solution is a result of the underlying diffusion coherence and not of some other effect? Indeed, performing tractography on a data set with completely random and incoherent diffusion would produce many fibers that look improbable, but may also produce some fibers that, by chance or due to the brain geometry or a limited resolution, look “real”. In this context, it is essential to identify and to quantify the source of error, not only for a specific tract, but for a whole brain tractography experiment.

A straightforward way to tackle this issue is to evaluate the effects of noise on the diffusion MR data. For example, several studies focus on the impact of noise on diffusion tensor eigenvalues, as well as on the derived fiber trajectories [15], [18], [21]. Others try to model the eigenvector dispersion by assuming various probability density functions [22], [23]. Another approach consists in measuring the uncertainty associated with the reconstructed fiber trajectories. Probabilistic algorithms particularly well suit this task, since they allow to assign a probability to the produced tracts, either by integrating a cost-function along the paths [8], or by counting the occurrence of the paths obtained using a Monte Carlo random walk [9], [10]. In contrast, deterministic algorithms suffer from the lack of information on the probability of the reconstructed trajectories. In [24], a bootstrap approach is proposed to estimate the dispersion associated with tractography results. Recently, this method was extended to use any deterministic tractography algorithm in a probabilistic way [25].

In this study, as in [25], we try to address the shortcomings of the deterministic approach. However, instead of transforming deterministic algorithms into probabilistic ones, we rather add some information about the reliability of the produced tracts. To this purpose, we present a method specifically aiming at differentiating connections likely to be built by diffusion coherence contrast from those potentially resulting from non-diffusion effects, such as noise, resolution or brain geometry. We proceed by comparing the fiber density of every connection with a set of equivalent connections generated in systems where diffusion coherence contrast is removed by randomly reshuffling the orientation distribution functions (ODFs). First, we study the statistical differences between connections in data sets with and without diffusion coherence contrast. We show that on an individual basis some connections are clearly different in the two types of data sets, while others cannot be differentiated. Then, we define a confidence level in order to quantify the difference between the data sets with and without diffusion coherence contrast. We show that the confidence level greatly varies from one connection to another. We analyze the factors responsible for this high variability and emphasize the fact that one cannot trust all fibers equally in tractography.

Finally, we propose a method to reduce the computation time of the estimation of the confidence level, based on the apparent correlation between confidence level and connection distance. This methodology is expected to add useful information to any tractography study, deterministic [26] or probabilistic [27].

## Materials and Methods

**Figure 1:**
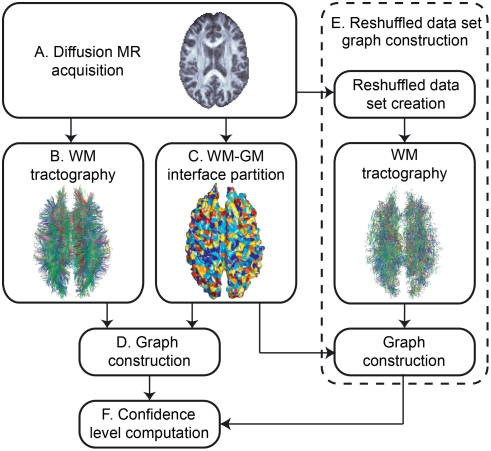
Overview of the whole process. Overview of the whole process. (A) Acquisition of the diffusion MR images. (B) Tractography in the brain WM. (C) Partitioning of the WM-GM interface into small regions of interest (ROIs). (D) Creation of the original brain connectivity graph using the results of steps B and C. (E) Construction of randomized versions of the original brain connectivity graph (the same partition into ROIs is used). (F) Computation of the confidence level of every edge in the original brain connectivity graph.

This research was conducted in agreement with the ethics comity for clinical research of the University of Lausanne (http://www.unil.ch/fbm/page36053.html) and informed written consent was obtained from the subjects before performing the study, in accordance with institutional guidelines. The proposed method consists of six steps, as described in Figure 1, and is identical to the method used in [26]. First, we acquire the diffusion MR images and process them in order to get a map of the diffusion in the brain (A). Next, we perform the tractography in the brain white matter (WM) (B). Independently from the tractography solution, we partition the WM-gray matter (GM) interface, i.e., the cortex for simplification, into small regions of interest (ROIs) (C). Once these steps are performed, we build a graph in which every ROI of the WM-GM interface constitutes a node. If there are some fibers linking a pair of ROIs, we build an edge between the corresponding nodes of the graph. That way, we obtain a graph reflecting the connectivity of the brain, that we call *graph of brain connectivity* (D). Next, we use a similar procedure (steps B to D) to construct randomized versions of the connectivity graph (the same partition into ROIs is used); the only difference is that now the diffusion map is randomized by reshuffling arbitrarily the ODFs. Finally, we compute the confidence level of the connections using the graphs derived from both the original and reshuffled data sets. We describe each of these steps below.

### A Diffusion MRI acquisition

The images from a diffusion MRI experiment of a human brain are acquired on four healthy volunteers with an Achieva 3T Philips scanner. We use a diffusion weighted single shot EPI spin echo sequence with the following timing parameters: TR/TE/Δ/δ = 4200/89/43.5/32.5 ms, where Δ is the diffusion time interval and δ the diffusion gradient duration [28]. With maximal diffusion gradient intensities of 80 mT/m this yields a maximal b-value of 9000 s/mm^2^. Q-space is sampled over a hemisphere using 129 different encoding gradients, and the data are reconstructed following a classical DSI scheme (see Material and Methods S1) [4], producing a 3D diffusion probability density function (pdf) in every voxel. The acquisition block is made of 36 slices of a 112×112 matrix, with a spatial resolution of 2×2×3 mm^3^. The acquisition time is approximately 18 minutes. Next, we simplify the data by computing for each voxel the ODF, a radial integration of the resulting diffusion pdf [4] (see Material and Methods S1). Moreover, a high resolution T1-weighted (MP-RAGE) MRI is performed on the same volunteer. This acquisition is then registered on the diffusion images using the affine registration method based on maximization of mutual information of Maes et al. [29], and used to identify both the WM and GM with a T1w-based segmentation algorithm [30], [31], which allows us to define the WM-GM interface.

### B White Matter tractography

DSI tractography is performed in WM using an algorithm especially designed for DSI data [13], [16], [32]. Note that any tractography algorithm can be used to generate the fibers, as long as it uses a deterministic approach. First, the ODF of each voxel is reduced into a set of normalized direction vectors corresponding to the local maxima of the ODF. Then, we choose a set of initialization points uniformly distributed in each brain WM voxel. The number of points is proportional to the number of direction vectors in the corresponding voxel. Next, from each of these points a fiber starts growing with a fixed step size (arbitrarily chosen to be 1 mm) in two opposite directions, locally following the direction of the diffusion maximum which is the closest to the fiber trajectory. If this results in a change of direction sharper than a fixed threshold (0.25 rad/mm), the fiber is stopped. The growing process ends when the end-points of the fiber have reached the WM-GM interface. Fibers that do not reach the WM-GM interface are eliminated. In this work, approximately 1 million fibers are generated in the brain WM. For more information about the tractography algorithm please see Material and Methods S1.

### C WM-GM interface partition into ROIs

The aim of this step is to partition the WM-GM interface into small ROIs. A simple approach would be to partition this interface according to a 3D regular grid. However, this method would result in regions with variable sizes, which is not acceptable, since we will work later with the density of fibers connecting ROIs. Moreover, we want the ROIs to be placed in the same anatomical location such that the connectivity can be compared locally.

The proposed procedure is based on an atlas-based cortical registration method using the curvature information, i.e. sulcus and gyrus [33], [34], [35]. This method has been directly implemented in the Freesurfer software (http://surfer.nmr.mgh.harvard.edu), which provides an automatic labelling of the cortex into 66 gyral-based parcels, which are defined using curvature-based information on 40 manually labelled brains [35]. The proposed procedure consists of three steps. First, we use Freesurfer to register a labelled mesh from an average brain onto the brain of each subject, where each label corresponds to one of the 66 anatomical regions, providing for every subject a standardized partition of the cortex into 66 anatomical cortical regions. Second, we subdivide each gyral-based parcel of the atlas into many small ROIs, in order to build a new atlas containing approximately one thousand ROIs. Finally, we register the obtained subdivision on the brain of each subject using the same transformation as for the 66 regional areas, thus maintaining the topological constraints of mapping. For more information on the partitioning process please see Material and Methods S1.

Using this procedure, we divided the cortex into 998 ROIs, compact and of similar size, and with a surface of about 140 mm^2^. An example of the obtained parcellation can be seen in Figure S1.

### D Construction of the original brain connectivity graph *G_O_*

In what follows, we will use the term *fiber* when referring to a single tractography fiber connecting two ROIs. The abstract link between two nodes in a graph will be denoted by the term *edge*.

We create the original brain connectivity graph *G_O_* by combining the output of the two previous steps (B and C) [32], [36], [16]. Every ROI becomes a node of the graph. We build an edge *e* between every pair of nodes and define its weight as follows:
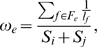
(1)


With *F_e_* the set of fibers contributing to the edge *e*, i.e., the fibers whose end-points lie in the corresponding ROIs (denoted by *i* and *j*), *l_f_* the length of the fiber *f*, and *S_i_*, *S_j_* the surface of the ROIs *i* and *j* respectively. If no fiber exists between two ROIs, a zero weight is assigned to the corresponding edge. The edge weight captures the fiber density between two nodes, in terms of number of fibers per unit surface. *l_f_* is a correction term needed to suppress the linear bias towards longer fibers introduced by the tractography algorithm [32]. Indeed, due to the initialization process which chooses a fixed number of fiber starting points per orientation in each voxel, the number of fibers generated along a given path is proportional to the length of this path. Therefore, we have to normalize the contribution of each fiber by its length when computing the edge weight. Finally, we introduce the edge distance *l_e_*, defined as the geodesic distance in the brain WM (i.e., the shortest path being confined in the WM mask) separating the two ROIs corresponding to the end-nodes of the edge in the graph.

### E Construction of the randomized brain connectivity graphs *G_Ri_*

Remember that we want to compare the original tractography solution with others where diffusion coherence contrast has been lost. This is achieved as follows. Starting from the original acquisition data set, we find and randomly reshuffle the voxels corresponding to the brain WM, i.e., we randomly permute the ODFs of these voxels. In other terms, this is a re-sampling of the data, or more particularly of the WM voxels, without replacement. Note that the reshuffling we perform is fundamentally different from the bootstrapping method used for example in [25]. Indeed, bootstrapping techniques are generally used in order to assess the accuracy of an estimator. In this work, we perform a re-sampling only in order to loose the diffusion coherence contrast in the data. It results in a data set whose geometrical properties, such as the WM tractography mask and the WM-GM interface, are identical to the original one. Similarly, the number and orientation of the main diffusion directions are preserved, but, what is crucial, the information about diffusion coherence is lost. We generate 30 reshuffled data sets (this choice will be discussed in the following). Next, we perform tractography to generate a solution on every reshuffled data set. Finally, as in D, we construct the connectivity graphs *G_Ri_* based on the randomized tractography results and the same partition into ROIs as obtained for the original data set (C).

### F Confidence level computation

Let *ω_e,O_* and *ω_e,Ri_* denote the weight of the edge *e* in *G_O_* and *G_Ri_*, respectively. Considering the set of randomized brain connectivity graphs 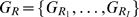, for every edge *e* we have a set of edge weights 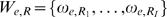 coming from a specific distribution. This set of edge weights *W_e,R_* provides us with an empirical distribution, which is an estimate of the true underlying distribution.

Let 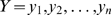 be some real data and let *p* be a proportion between 0 and 1. A quantile 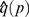 is a value such that a proportion *p* of the observations *Y* is smaller than 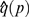. In our case, *Y* is replaced by the set of edge weights in *G_R_*, that is *W_e,R_*. The edge weight in *G_O_* can be interpreted as a quantile of *W_e,R_*, with 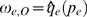. We define the confidence level *c_e_* as the proportion of *W_e,R_* being smaller than *ω_e,O_*, that is 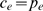.

The confidence level is computed for every edge whose weight in *G_O_* is strictly positive (called a non-zero edge in the following). The maximum for the confidence level is the unity, it indicates that *ω_e,O_* is higher than all the values in *W_e,R_*. Similarly, the minimum is zero and means that *ω_e,O_* is lower than all the values in *W_e,R_*. It is important to note that the confidence level is computed for each edge, independently from the other edges in the graph.

## Results

This section is divided in four parts. In the first one, we compare the original brain connectivity graphs *G_O_* with a set of randomized equivalents *G_R_*, in terms of node degree, node strength, number of connections, edge distance and edge weight, hence analyzing the structural differences between these two types of data sets. Next, we analyze, with the help of the constructed confidence level, the contribution of the diffusion coherence contrast to every connection. In the third part, we show as an illustration how to integrate the confidence level into the connectivity matrix. Finally in the last part, introduce a way to optimize the computation.

### A Comparison of *G_O_* with *G_R_*

In what follows, the plots compare the original graph *G_O_* with a single randomized brain connectivity graph *G_Ri_*. Since the variations observed among the set of randomized graphs *G_R_* are not significant (data not shown), these plots are valid for the set of randomized graphs *G_R_*. Therefore, we will speak of *G_Ri_* to refer to any single randomized brain connectivity graph. It is also important to note that all these experiments have been performed for one subject at three different scales of the connectivity graph: 500, 1000 and 2000 nodes. As the results were similar for the three scales, we decided to only work with the graphs with 1000 nodes.

**Figure 2:**
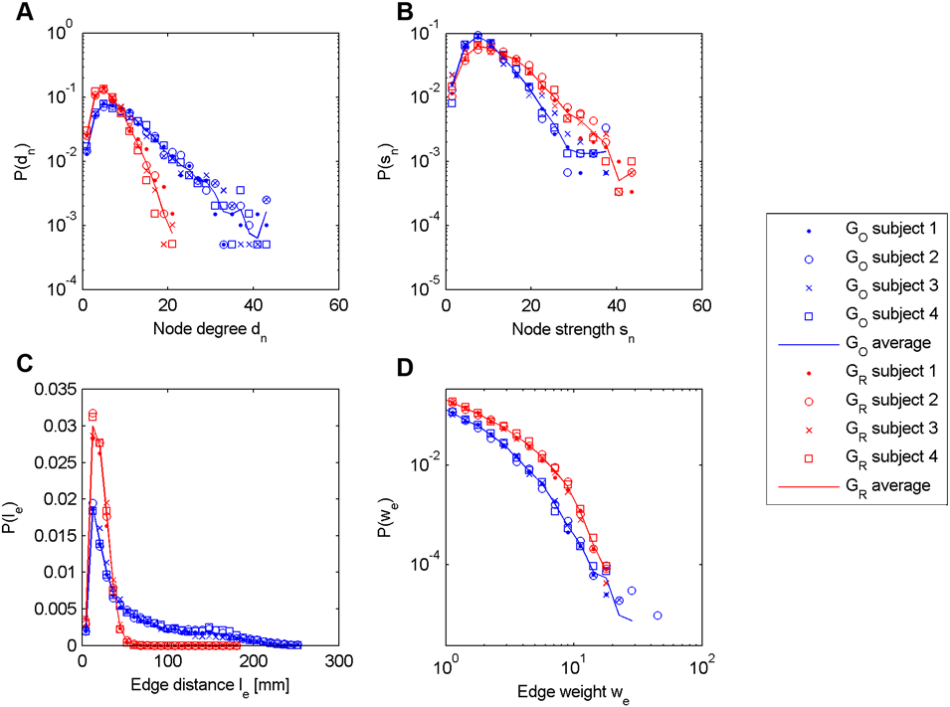
Node and edge statistics for *G_O_* and *G_R_*. Node and edge statistics for *G_O_* and *G_R_*. A: Node degree distribution. B: Node strength distribution. C: Edge distance distribution. D: Edge weight distribution.

In order to compare the original and randomized graphs, we first turn our attention to the nodes of the graphs. We focus on two basic node characteristics: the degree *d_n_*, i.e., the number of edges incident on the node *n*, and the strength *s_n_*, which is the sum of weights of all edges incident on the node *n* [37]. In Figure 2, we report the node degree (A) and node strength (B) distributions for both *G_O_* and *G_Ri_*. We also look at two edge statistics: the edge distance *l_e_* distribution (C) and the edge weight *ω_e_* distribution (D) (computed on non-zero edges only). We can see that *G_O_* presents a node degree distribution with a heavier tail than *G_Ri_*, indicating that some nodes are more connected in the original brain connectivity graph. This is explained by the fact that the number of connections is higher in *G_O_* (9926 edges in average) than in *G_Ri_* (5686 edges). In Figure 2C, we can see that the edge distance is much shorter in *G_Ri_* than in *G_O_*, indicating a loss of long-range connectivity in *G_Ri_*, which explain this difference in the number of connections. In Figure 2B, we can see that there are slightly more nodes with a high node strength in *G_Ri_* than in *G_O_*. This can be explained by the fact that the edge weight is higher in *G_Ri_* than in *G_O_*, as shown in Figure 2D. A more complete evaluation of these brain connectivity graphs can be found in [32].

**Figure 3:**
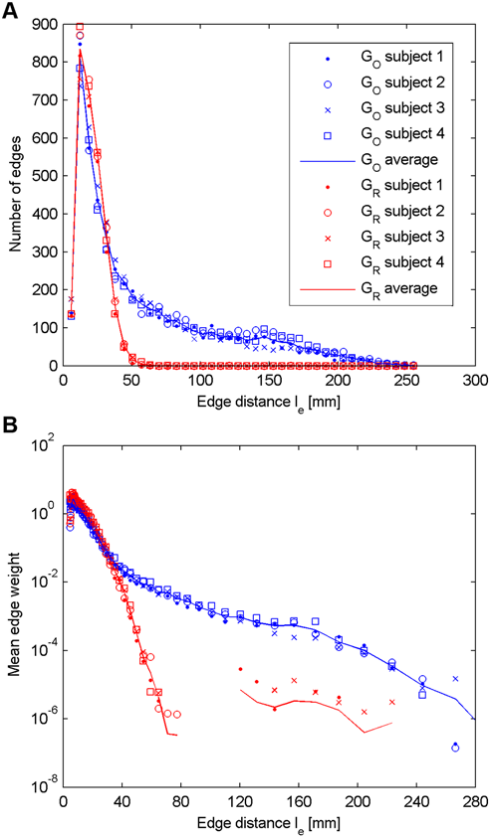
Distribution of non-zero edges and mean edge weight vs. edge distance. (A) Distribution of non-zero edges in *G_O_* and *G_R_* as a function of the edge distance *l_e_*. (B) Mean edge weight in *G_O_* and *G_R_* as a function of the edge distance *l_e_*.

Next, we focus on the evolution of the connectivity with respect to the edge distance. In Figure 3A, we plot the number of non-zero edges as a function of the edge distance. Figure 3B represents the mean edge weight as a function of the edge distance. These plots lead us to the following observations.

#### a) Short connections

The tractography algorithm produces quite accurately the same number of short non-zero edges (typically below 40 mm) in both *G_O_* and *G_Ri_* (Figure 3A), and the mean edge weight for edges of short edge distance is almost identical (Figure 3B). Since a fiber between two given ROIs is found by the tractography algorithm only if a path of coherently aligned directions of maximal diffusion exists, we deduce that for two closely located ROIs the chance of finding such a path of coherent diffusion in the reshuffled data set is non negligible. It is also important to note that for short connections the standard deviation of the edge weight is almost identical in both *G_O_* and *G_Ri_*, and the edge weight distribution for a given edge distance is similar in both cases (data not shown). Furthermore, in Figure 3B, in the short range, there seems to be a clear dependence between mean edge weight and edge distance.

#### b) Mid- and long-range connections

Unlike for short connections, it seems that for mid- and long-range connections some fundamental differences appear between the original and the reshuffled data sets. We remember that the tractography is run in exactly the same way and with the same parameters in all cases (particularly with the same number of fiber initializations). However, we see that the original brain connectivity graph *G_O_* contains more non-zero edges than *G_Ri_*, and that these additional edges are mid- and long-range. Similarly as in the short range, the mean edge weight in *G_Ri_* continues to decrease with increasing distance, following an exponential law. This is because when the edge distance *l_e_* increases, the chance of finding a path of coherent diffusion sharply decreases. On the other hand, the mean edge weight in *G_O_* changes its behavior at around an edge distance of 40 mm by decreasing very slowly with increasing edge distance. The fiber density (i.e. edge weight) of an edge in *G_O_* seems thus not to be dependent from its distance. Interestingly, it turns out that the limit of 40 mm is consistent across the subjects and is not dependent on the scale of the connectivity graphs, since similar results were found for graphs with 500, 1000 and 2000 nodes (data not shown), but might change with other parameters as we discuss below.

### B Confidence level analysis

In this part, we focus on the analysis of the confidence level. Remember that every non-zero edge obtained in the original brain connectivity graph *G_O_* has a confidence level, that depends on two factors: (i) its edge weight in the original data set *ω_e,O_*, and (ii) the set of weights obtained for the same edge *e* in the randomized brain connectivity graphs 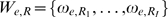. This measure is thus independent from the other edges of the graph, and is to some extent independent from the brain geometry, since the position of the nodes is exactly the same in both *G_O_* and *G_R_*.

**Figure 4:**
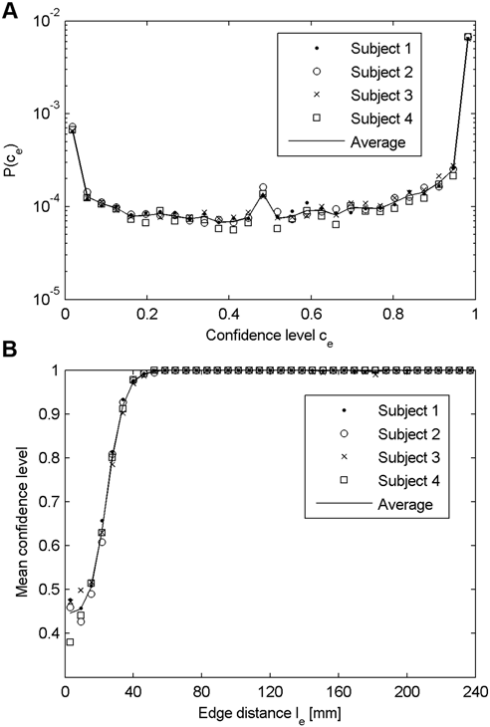
Confidence level distribution and mean confidence level vs. edge distance. (A) Distribution of the confidence level computed for non-zero edges. (B) Mean confidence level as a function of the edge distance *l_e_*.

First, we show in Figure 4A the distribution of the confidence levels. According to the definition of the confidence level, a zero value 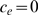 means that the edge has a lower weight than all the realizations of the same edge in *G_R_*. On the other hand, 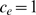 corresponds to edges whose weight is higher than all the realizations of the same edge in *G_R_*. In the middle range, 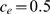 indicates that the edge weight obtained in *G_O_* is comparable to the median of the measures obtained in *G_R_*. We can see in Figure 4A that most of the distribution is close to the maximum value, meaning that the tractography globally produces tracts that are the result of the measured diffusion coherence contrast. However, the peak near the minimum value indicates that some of the edges produced by the tractography have a stronger weight in *G_R_* than in *G_O_*, and are thus probably due to non-diffusion effects.

Next, we investigate the evolution of the confidence level with respect to the edge distance. In Figure 4B, we plot the mean confidence level as a function of the edge distance. We can see that the confidence level rapidly increases with the edge distance. From a distance of around 60 mm the mean confidence level reaches the maximum value and stays constant. We can then say that short edges globally present a low confidence level. In contrast, we can have a strong confidence in mid- and long-range connections.

### C Example of application

**Figure 5:**
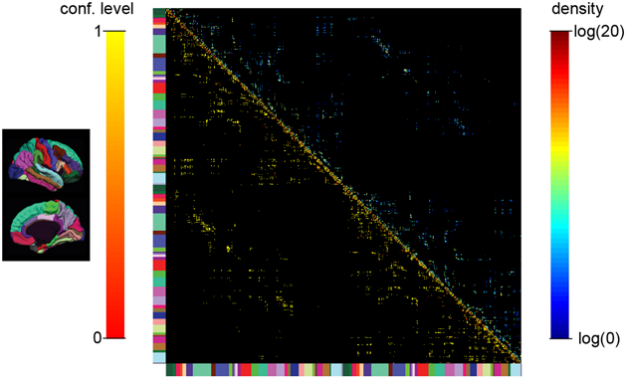
High-resolution structural connection matrix. High-resolution structural connection matrix, representing the fiber density (upper triangular part) and the confidence level (lower triangular part). The matrix is organized as follows: the upper left block represents the connections in the right hemisphere and the lower right block shows the connections in the left hemisphere. The off-diagonal blocks map the inter-hemispheric connections. The color bar at the left and bottom of the matrix help to make the correspondence between the matrix entries and the cortical parcels as displayed on the left part of the figure.

A representative example of a high-resolution structural connection matrix of an individual human brain is shown in Figure 5. The matrix is organized as follows: the upper left block represents the connections in the right hemisphere and the lower right block shows the connections in the left hemisphere. The off-diagonal blocks map the inter-hemispheric connections. The color bar at the left and bottom of the matrix help to make the correspondence between the matrix entries and the cortical parcels as displayed on the left part of the figure. Since the connections are not oriented the matrix is symmetric. Therefore, we can show simultaneously two parameters for every connection in the matrix. The upper triangular part of the matrix represents fiber densities between pairs of single ROIs. The lower triangular part shows the confidence levels associated to the connections. The confidence level which is added in this matrix is a valuable help towards the interpretation and the assessment of the whole brain connectivity. We can see that the connections which present a low confidence level are mainly located very close to the diagonal, which corresponds to intra-parcel connections. This is not surprising since those connections are mainly composed of short fibers. On the contrary, longer connections such as the inter-hemispheric connections (off-diagonal blocks of the matrix) present a very high confidence level.

### D Optimization of the computation

As mentioned in the methodology, the confidence level introduced in this work is based on the set of edge weights obtained with *G_R_*, that is 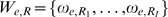. For a given edge *e*, the more samples we have in *W_e,R_*, the better the estimation of the true underlying distribution, and thus the more accurate the confidence level. Therefore, one would be tempted to create a lot of randomized brain connectivity graphs, but unfortunately their generation is computationally very expensive. In this context, there is a need for optimization of the computation.

In Figure 3B we see that the mean edge weight in *G_Ri_* depends on the edge distance *l_e_*. Let us suppose for a while that the other factors, such as the brain geometry or the tractography algorithm, do not significantly influence the mean edge weight in *G_Ri_*, or at least affect the edges uniformly regardless of the edge distance *l_e_*. In this case, the sets of edge weights 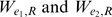 produced by two different edges, *e_1_* and *e_2_* with the same edge distance *l_e_*, are sampled from the same underlying distribution. Therefore, by grouping the edges according to their edge distance *l_e_*, we increase the number of samples that constitute the empirical distributions of the edge weights. From a practical point of view, this means that instead of generating 30 reshuffled data sets which is very time-consuming, producing for each edge *e* a set of 30 edge weights *W_e,R_*, we create only one reshuffled data set. Next, we group the edge weights having a similar edge distance *l_e_* and create sets of edge weights dependent on the edge distance only. Denote by *W_d,R_* the set of all edges whose edge distance *l_e_* is close to *d*, that is 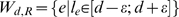, with *ε* a tolerance value. The confidence level is then computed as described above, by replacing *W_e,R_* with *W_d,R_*.

**Figure 6:**
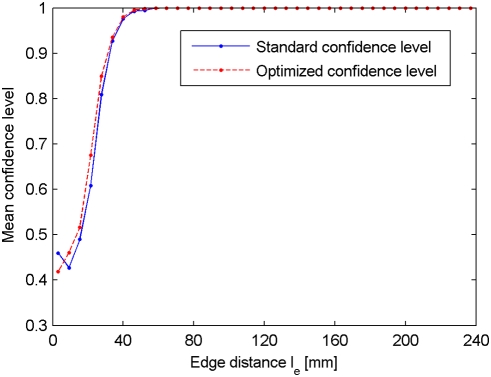
Standard vs. optimized confidence levels: mean confidence level vs. edge distance. Mean standard and optimized confidence levels as a function of the edge distance *l_e_*.

**Figure 7:**
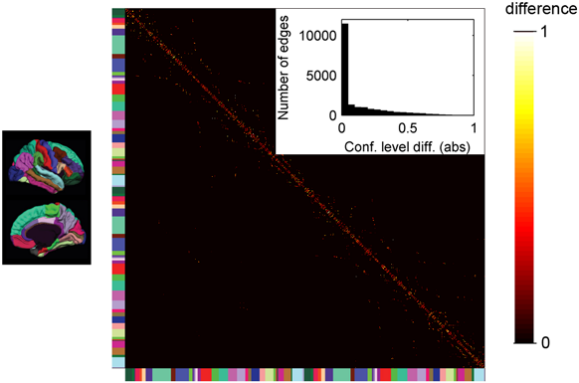
Matrix of the differences between standard and optimized confidence level. High-resolution matrix, representing the difference between the standard and optimized confidence level (absolute values). In the insert: distribution of the differences between standard and optimized confidence level (absolute values), computed for non-zero edges.

We compare the confidence level computed on the set of 30 reshuffled data sets *G_R_*, called standard confidence level, with the proposed method, denoted by optimized confidence level. In Figure 6 we report the mean confidence level as a function of the edge distance, for both methods and for a single subject (the results obtained with the other subjects are similar, data not shown). It turns out that the mean confidence level is very similar for both the standard and the optimized methods. This observation is confirmed by a correlation of 0.79 (averaged over the four subjects) between the standard and optimized confidence levels. In Figure 7, we report the matrix containing the absolute value of the differences between the two confidence levels. We can see that the difference is only rarely higher than 0.2, which is confirmed by a distribution very close to zero, as shown in the insert of Figure 7.

## Discussion

### A The rational behind the method

How can we be sure that a given computed connection is due to diffusion coherence contrast (i.e., underlying neuronal anatomy) and not to noise, limited resolution (aliasing), brain geometry or to the properties of the tractography algorithm itself? In other words, how can we know that a given image feature (i.e., a connection) comes from a specific physical process (diffusion) and not from other uncontrolled imaging parameters? Given that we have an adequate image model, the straightforward method is to study the behavior of the imaging feature in an identical data set from which the specific physical process has been removed (i.e. in which the diffusion coherence contrast is removed). In practice, we can obtain such a data set by randomly reshuffling the voxels in the WM mask with their associated ODFs. Then, we can perform the tractography and compute the associated connectivity graph. By creating a large number of reshuffled data sets, we can finally compute the confidence level of a given edge in *G_O_* with respect to the estimate of the weight distribution of the same edge obtained in *G_R_*. This method provides us with a measure of how unlikely it is that the measured edge weight comes from something else than diffusion coherence contrast.

However, we have to keep in mind that tractography is a method that maps lines of diffusion coherence inside the white matter. The relation between real or anatomical fibers and pseudo-fibers produced by the tractography is conceptual and experimental to some extent [14], [19]. Consequently, the confidence level does not give information about the existence of fibers. Instead, as mentioned in [25], it refers to the “amount of confidence we can place in the tract realization not being a spurious *one off* occurrence that has been unusually corrupted by noise, motion, or other sporadic artifacts”. We also insist on the fact that the confidence level does not improve the tractography quality. It just gives additional information about the tractography solution in order to help its interpretation.

One of the issues of this work is related to the number of reshuffled data sets. Indeed, the more reshuffled data sets we produce, the more accurate the empirical quantiles, and thus the more accurate the confidence level. This raises the following question: Are the 30 reshuffled data sets used in this work sufficient to have an accurate estimate of the confidence level? Since the reshuffled data sets are generated independently, the samples constituting the set of edge weights *W_e,R_* are also independent. This means that the empirical distribution provided by *W_e,R_* is an unbiased estimate of the true underlying distribution. The confidence levels are thus not biased by the number of reshuffled data sets. Consequently, increasing the number of data sets would of course improve the accuracy of the confidence level, but would not significantly modify the results we obtained. Finally, we should insist on the fact that generating a reshuffled data set is very time-consuming. Thus, generating more than 30 data sets is practically not reasonable.

### B The effect of the spatial resolution

When we consider the relation between the mean edge weight and the edge distance in the original data set (Figure 3B), we notice that it is bimodal. In the range of short distances (below 40 mm), the plot follows a similar behavior as the reshuffled data set, while for longer distances the mean edge weight decreases very slowly. The relative constancy of the mean edge weight with respect to the edge distance is a very important observation. It tells us that our way of measuring connectivity is not biased by distance. The initial bump for edges shorter than 40 mm may be explained in two ways. First, we know that the brain performs most communication locally (functionally associated cortical areas are nearby) [38], [39], yielding possibly stronger connectivity in a short range. Second, which is in our opinion the most likely explanation, is that for short-range edges there is no big difference between principal diffusion orientations that are coherently versus randomly oriented given the small number of tracking steps. This difference is a consequence of a low spatial resolution. Indeed, the shorter the distance between two distinct ROIs, the lower the number of voxels separating these ROIs, and thus the higher the probability of finding a path of coherent diffusion linking these ROIs by chance. Therefore, the only way to keep track of the short-range connections would be to increase the spatial resolution of the diffusion MRI acquisition, which would in turn decrease the chance of connection at random. This also means that we potentially overestimate the number of short connections due to the additional effect of true and “by chance” diffusion coherence over short distances.

Considering Figure 3B, one may be tempted to consider the ratio between the mean edge weight in the original and the reshuffled data sets as a measure of the signal to noise ratio (SNR). Presented that way, we directly notice that the SNR is too low for fibers below 40 mm to make any statement. In particular, a low confidence level in these connections does not mean that they do not exist, but only that they cannot be faithfully distinguished from connections created by the unpredictable effects.

### C Optimized versus standard confidence level

The optimized confidence level is based on the assumption that the mean edge weight in *G_R_* depends mainly on the edge distance *l_e_*. The other factors, such as the brain geometry or the tractography algorithm parameters, are supposed to have only a minor influence on the edge weight, or at least to affect the edges uniformly regardless of *l_e_*. Of course, this assumption is very restrictive, and practically not really verifiable. However, the similar results produced by both the standard and the optimized method seem to confirm the hypothesis. We do not pretend that the brain geometry does not play a role on the edge weight, but we believe that its effect is limited. Thus, we think that the optimized confidence level is an adequate method to dramatically reduce the computation time, although more investigations have to be performed to confirm our results.

As stated before, the probability of finding a path of coherent diffusion is rather low in a reshuffled data set, and therefore a large proportion of edges in *G_R_* have a zero weight. Due to the limited number of reshuffled data sets, the confidence level of long-range connections tends to be slightly over-estimated. Apart from the computation time, another advantage of the optimized confidence level is that the grouping of edges increases the number of samples per estimated distribution. Consequently, the optimized confidence level partially solves the issues raised by the limited number of reshuffled data sets.

### D Advantages, drawbacks and future work

In this article we present a method to associate a confidence level with a connection measured with tractography. This confidence level allows us to quantify the contribution of the diffusion coherence contrast to the produced tracts. We observe that tractography maps well the diffusion coherence contrast over long distances but that for short-range trajectories it is impossible to say whether their source is the diffusion coherence or chance. The direct consequence is important for all studies that aim at mapping and characterizing short-range connections; their results should be interpreted with enormous care.

However, it is worthwhile to point out that very important contributors to aberrant connectivity mapping are not filtered out with the presented methodology. Indeed, even if the diffusion MRI experiment is performed properly, i.e. ideally without susceptibility artifacts or other systematic biases, there are in our opinion, two sources of errors: noise and aliasing or insufficient resolution. The effect of noise is rather straightforward: it produces unwanted principal directions of diffusion, yielding aberrant and missing connections [24], [25]. The question of aliasing is more difficult to analyze and would justify an article on its own. Schematically, insufficient angular and spatial resolutions yield 1) biased principal directions of diffusion (e.g. smoothing of two diffusion peaks into one) 2) partial volume effects [40] which can be the cause of constructing aberrant fiber tracts. Indeed, when the spatial resolution is insufficient relatively to the maximal curvature radius of fiber bundles, tracts that cross in reality may kiss in the tractography reconstruction and vice versa, thus creating aberrant solutions.

The confidence level computed in this work is based on the edge weight only. However, it is highly likely that other features could help in the evaluation of short-range connections. A possible way to improve the quality of the confidence level would be to include a measure of the dispersion of the trajectories, as follows. Let us consider an edge connecting two close ROIs. In the reshuffled data sets the fibers constituting this specific edge should present various trajectories, which can be captured by a high variability of the fiber length. In contrast, in the original data set, if a path of maximal diffusion exists between the two ROIs, most of the fibers constituting the corresponding edge should roughly have the same trajectory, and therefore have the same length. Consequently, the variance of the fiber length could help in the evaluation of the confidence level of short-range connections.

### E Conclusions

In this work, we propose a method to evaluate the confidence level of every connection obtained by tractography, in order to discriminate the fibers resulting from the diffusion signal itself from those due to some non-diffusion effects. According to the presented results we can say that the tractography, as it is performed in this work, is well suited to map mid- and long-range connections with a high confidence level. On the contrary, the low confidence level found for the short-range connections indicates that some precautions must be taken when mapping the brain short-range connectivity. In our opinion spatial resolution is one of the main factors that affect the accuracy of short-range connections in tractography.

## Supporting Information

Material and Methods S1 (0.07 MB DOC)

Figure S1 (A) Standardized partition of the cortex into 66 cortical regions. (B)Example of the parcellation of the cortical regions on the atlas. (C) Same parcellation as in B, after the registration on the subject's cortex.
(0.32 MB TIF)
