# Supplementary material for: Estimating the Confidence Level of White Matter Connections Obtained with MRI Tractography
Source: PLoS One. 2008 Dec 23;3(12):e4006. doi: 10.1371/journal.pone.0004006 (PMC2603475; doi:10.1371/journal.pone.0004006)
Supplement: Material and Methods S1 — (0.07 MB DOC) [file pone.0004006.s001.doc]

### **Material and Methods S1**

### A Diffusion MRI acquisition

Five data sets are acquired at 3T with an Achieva (Philips, Einthoven, The Netherlands) MRI scanner using a diffusion weighted spin echo EPI technique [1, 2]. The timing parameters of the pulse sequence are TR/TE/Δ/δ = 4200/89/43.5/32.5 ms, maximum diffusion gradient intensity is 80 mT/m, yielding a maximal b value of 9000 s/mm^2^ [3]. The matrix size is 112x112 and the slice number is 36, with a voxel size of 2x2x3 mm^3^. Diffusion imaging is performed according to the classical Diffusion Spectrum Imaging (DSI) scheme as described in [3]. Briefly, diffusion-weighted images covering the whole brain are acquired for 129 different values of diffusion sensitizing gradient intensity and direction (i.e., different q-vectors) [4], comprising in q-space the points of a cubic lattice within a hemisphere of 4 lattice units in radius. We take $\mathbf{q}=a\mathbf{q}_{x}+b\mathbf{q}_{y}+c\mathbf{q}_{z},$with $a,b,c$ integers and $\sqrt{a^{2}+b^{2}+c^{2}}\leq4$, and$\mathbf{q}_{x},\mathbf{q}_{y}$,$\mathbf{q}_{z}$ denoting the unit diffusion sensitizing gradient vectors in the three respective coordinate directions. Next, we process these 129 images as follows. First, we reconstruct the 3D diffusion function, or Probability Density Function (PDF) at each brain location by taking the discrete 3D Fourier transform of the signal modulus sampled in q-space. The signal is pre-multiplied by a Hanning window before Fourier transformation in order to ensure a smooth attenuation of the signal at high $|\mathbf{q}|$ values. With this procedure and the above parameters the PDF is sampled over an isotropic 3-dimensional field of view of 100μm with a nominal isotropic resolution of 10μm. The result, called a *diffusion map*, is an image that associates a 3D diffusion function with every brain position voxel. From this map, at each voxel, we compute an Orientation Density Function (ODF) *φ*(**u**), by projection of the PDF in the radial direction. For **u** a 3D vector with |**u**| = 1, we define:

$\varphi(\mathbf{u})=\int p(\rho\mathbf{u})\rho^{2}d\rho$ ()

where *p*(.) is the 3D PDF, *ρ* is the radius, *ρ ^2^dρ* is the 3D volume element and the integral is evaluated as a discrete sum over the available range *ρ = [0,4]*. The ODF *φ*(**u**) is a function defined on a discrete sphere and captures the diffusion “intensity” in every direction. It is evaluated for a set of vectors **u***_i_* that are the vertices of a tessellated sphere that has a mean nearest-neighbor separation about 10°.

### B White Matter tractography

We use a tractography algorithm specifically designed for DSI data. This method is described in [5] and summarized below:

1. At each voxel, we define a set of directions of maximum diffusion as local maxima of *φ*(**u**) (i.e., vectors **U***_i_* such that *φ*(**u***_j_*) *< *(**U***_i_*) for all **u***_j_* adjacent to **U***_i_* in the sampled tessellated sphere.
2. Fiber computation. We initiate the same number of fibers for every direction of maximum diffusion in every white matter voxel. For example in a voxel with 3 directions we initiate 25 fibers along each direction, total 75. The starting points are chosen at random within the voxel. Next, from each of such point we start growing a fiber in two opposite directions with a fixed step of 1mm. On entering a new voxel, the fiber growth continues along the direction of the vector **U***_j_* (in the new voxel) whose orientation is the closest to the current direction of the fiber. If this results in a change of direction sharper than 0.25 radian/mm, the fiber is stopped. The growth process of a valid fiber finishes when its both ends leave the white matter. The resulting fibers can be interpreted as an estimate of the white matter axonal bundle trajectories

C WM-GM interface partition into ROIs

The WM-GM interface partitioning is an important part of the processing, with several constraints. First, we want the ROIs to be as small and as compact as possible. Since we believe that current in-vivo tractography methods are accurate up to roughly a centimeter and that the performance of our partition technique is likely similar we have chosen to set the highest resolution to about 1000 ROIs corresponding to a ROI size of 1.5cm2. This is highly reasonable since it is comparable to the size of the smallest of the Brodmann areas. Second, we want the ROIs to be placed in such a way that the anatomical location stays constant among the different subjects.

The proposed procedure is based on an atlas-based cortical registration method using the curvature information, i.e. sulcus and gyrus [6, 7]. This method has been directly implemented in the Freesurfer software ([http://surfer.nmr.mgh.harvard.edu](http://surfer.nmr.mgh.harvard.edu/)), which provides an automatic labelling of the cortex into 66 gyral-based parcels, which are defined using curvature-based information on 40 manually labelled brains [7]. The proposed procedure consists of three steps. First, we use Freesurfer to register a labelled mesh from an average brain onto the brain of each subject, where each label corresponds to one of the 66 anatomical regions, providing for every subject a standardized partition of the cortex into 66 anatomical cortical regions (see Figure S1A). Second, we subdivide each gyral-based parcel of the atlas into many small ROIs, in order to build a new atlas containing approximately one thousand ROIs (see Figure S1B). Finally, we register the obtained subdivision on the brain of each subject using the same transformation as for the 66 regional areas, thus maintaining the topological constraints of mapping (Figure S1C).

a) Partition of the atlas into one thousand ROIs

First, we determine the number of ROIs per parcel, denoted by Np, according to the relative surface of each template parcel as well as the desired total number of ROIs. This will ensure a minimal variation of the ROI surface between different parcels. Then, for each parcel p, a ROI grows from a randomly chosen point of p by aggregating the neighbouring voxels, until it reaches the desired surface, i.e. the total cortex surface divided by the desired number of ROIs. Next, other ROIs are generated from starting points located close to the already existing ROIs, until the parcel is fully covered with ROIs. In the second phase, we identify the Np biggest ROIs of the parcel and compute their centers of gravity. Then, again, we start a growing process from these points, but this time with all ROIs growing simultaneously. Using this two-phase partitioning heuristic, we obtain ROIs that are compact and of similar size [8]. Since we force the template ROIs to reach the same size, the resulting number of ROIs generated on the template cortex is actually 998 and not 1000. It is also important to emphasize that each parcel is subdivided individually, thus preventing the ROIs from belonging to different parcels. In that way, we force the ROIs to remain in the same anatomical region for different subjects. An example of this parcellation can be seen in Figure S1B.

b) Cortical registration

A brief description of the parcellation method [6, 7] implemented in the Freesurfer software follows. After some intensity normalization and re-sampling steps, the skull is removed from the T1 weighted image and the image is segmented, to identify the dorsal, ventral and lateral extent of the grey/white matter boundary to provide a surface representation of the cortical white matter [9-11]. The surface depicting the interface between cortex and white matter is automatically corrected for topological defects [12], and thereafter used in a deformation procedure to locate the pial surface of the brain [13].

After closing the surface medially across sub-cortical structures, the cortical surface of one hemisphere is topologically equivalent to a sphere. The reconstructed hemispheric surface of the individual subject is inflated to determine the large-scale folding patterns of the cortex and transformed into a sphere in a manner that minimizes metric distortion [14]. A spherical surface-based coordinate system is established. The cortical surface is then divided into neuro-anatomical labelled regions with a spherical statistical atlas. This procedure models the labelling system as a first order anisotropic non-stationary Markov random field on the curvature of the cortical surface, allowing it to capture the spatial relationships and the variance between regions present in the training set [15, 6].

## References

[1] Stejskal E, Tanner J (1965) Spin diffusion measurements - spin echoes in presence of a time-dependent field gradient. J Chem Phys 42:288.

[2] LeBihan D (1991) Molecular diffusion nuclear magnetic resonance imaging. Magn Reson Q 7(1):1-30.

[3] Wedeen V J, Hagmann P, Tseng W-Y, Reese T, Weisskoff R (2005) Mapping complex tissue architecture with diffusion spectrum magnetic resonance imaging. Magn Res Med 54: 1377–1386.

[4] Callaghan P T (1991) Principles of Nuclear Magnetic Resonance Microscopy. Oxford science publications XVII:492-494.

[5] Hagmann P, Reese T, Tseng W-Y I, Meuli R, Thiran J-P et al. (2004) Diffusion spectrum imaging tractography in complex cerebral white matter: An investigation of the centrum semiovale. Proc Intl Soc Mag Reson Med 12.

[6] Fischl B, Van Der Kouwe A, Destrieux C, Halgren E, Segonne E et al. (2004) Automatically parcellating the human cerebral cortex. Cereb Cortex 14(1): 11-22.

[7] Desikan R S, Segonne F, Fischl B, Quinn B T, Dickerson B C et al. (2006) An automated labelling system for subdividing the human cerebral cortex on MRI scans into gyral based regions of interest. Neuroimage 31(3): 968-80.

[8] Hagmann P, Kurant M, Gigandet X, Thiran P, Wedeen V J et al. (2007) Mapping human whole-brain structural networks with diffusion MRI. PloSONE 2:7.

[9] Dale A M, Sereno M (1993) Improved localization of cortical activity by combining EEG and MEG with MRI surface reconstruction: a linear approach. J. Cogn. Neurosci. 5:162-176.

[10] Dale A M, Fischl B, Sereno M (1999) Cortical surface-based analysis. I. Segmentation and surface reconstruction. Neuroimage 9(2): 179-94.

[11] Fischl B, Sereno M, Dale A M (1999) Cortical surface-based analysis. II: Inflation, flattening, and a surface-based coordinate system. Neuroimage 9(2): 195-207.

[12] Segonne F, Grimson E, Fischl B (2005) A genetic algorithm for the topology correction of cortical surfaces.Inf Process Med Imaging 19: 393-405.

[13] Fischl B, Dale A M (2000) Measuring the thickness of the human cerebral cortex from magnetic resonance images. Proc Natl Acad Sci U S A 97(20): 11050-5.

[14] Fisch B, Dale A M, Sereno M, Tootell R B H, Rosen B R (1998) A coordinate system for the cortical surface. NeuroImage 7:S740.

[15] Fischl B, Salat D H, Busa E, Albert M, Dieterich M et al. (2002) Whole brain segmentation: automated labelling of neuroanatomical structures in the human brain. Neuron 33(3): 341-55.
