# Supplementary figures and images for: Estimating the Confidence Level of White Matter Connections Obtained with MRI Tractography
Source: PLoS One. 2008 Dec 23;3(12):e4006. doi: 10.1371/journal.pone.0004006 (PMC2603475; doi:10.1371/journal.pone.0004006)

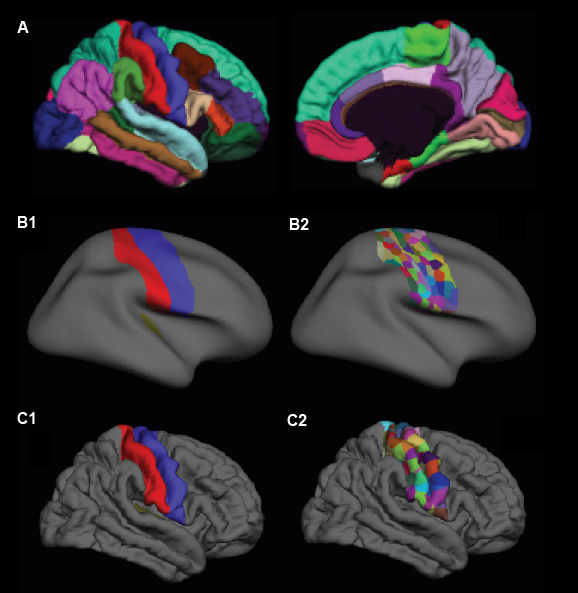

Supplement: Figure S1 — (A) Standardized partition of the cortex into 66 cortical regions. (B)Example of the parcellation of the cortical regions on the atlas. (C) Same parcellation as in B, after the registration on the subject's cortex. (0.32 MB TIF) [file pone.0004006.s002.tif]
